# Supplementary material for: Radiogenomics Map-Based Molecular and Imaging Phenotypical Characterization in Localised Prostate Cancer Using Pre-Biopsy Biparametric MR Imaging
Source: Int J Mol Sci. 2024 May 15;25(10):5379. doi: 10.3390/ijms25105379 (PMC11121591; doi:10.3390/ijms25105379)
Supplement: Supplementary file 1 [file ijms-25-05379-s001.zip › ijms-2986777-supplementary.pdf]

Supplementary Table S1: 22 GLCM (Gray-Level Co-occurrence Matrix) parameters and 6 histogram parameters for each Region of Interest (ROI).

Parameter	Description
GLCM Energy	Measure of texture uniformity; higher values indicate more homogeneity
GLCM Contrast	Measure of local intensity variation; higher values indicate more texture contrast
GLCM Homogeneity	Measure of closeness of the distribution of elements in the GLCM; higher values indicate more homogeneous textures
GLCM Correlation	Measure of linear dependency between pixel values at different locations in the image
GLCM Entropy	Measure of randomness in the texture; higher values indicate more randomness
GLCM Dissimilarity	Measure of average difference in intensity between pairs of pixels
GLCM Angular Second Moment (ASM)	Same as GLCM Energy, measures the uniformity of the image texture
GLCM Maximum Probability	Maximum probability of occurrence for a given gray-level pair
GLCM Inverse Difference Moment (IDM)	Measures local homogeneity, with higher values indicating more uniformity
GLCM Sum Average	Mean gray-level value of all co-occurring pairs of pixel values with the same sum
GLCM Sum Variance	Measures the variance of gray-level sums over all co-occurring pairs
GLCM Sum Entropy	Measures the amount of information or uncertainty in the sum average matrix
GLCM Difference Variance	Measures the variance of differences between neighbor pixels
GLCM Difference Entropy	Measures the amount of information or uncertainty in the difference matrix
GLCM Information Measure of Correlation 1 (IMC1)	Measures the correlation between the probability distributions of rows and columns of the GLCM matrix
GLCM Information Measure of Correlation 2 (IMC2)	Measures the similarity between two information patterns
GLCM Autocorrelation	Measures the degree of similarity between an image and itself over different distances and angles
GLCM Cluster Prominence	Measures the skewness and asymmetry of the GLCM, higher values indicate more asymmetry
GLCM Cluster Shade	Measures the asymmetry of the GLCM, higher values indicate more asymmetry
GLCM Sum Mean	Measures the mean of the sum values in the GLCM matrix
GLCM Sum Variance	Measures the variance of the sum values in the GLCM matrix
GLCM Sum Entropy	Measures the entropy of the sum values in the GLCM matrix
Histogram Mean	Average intensity value in the histogram
Histogram Variance	Measure of the spread of the histogram values around the mean
Histogram Skewness	Measure of the asymmetry of the histogram distribution
Histogram Kurtosis	Measure of the 'tailedness' of the histogram distribution

Parameter	Description
Histogram Energy	Measure of the uniformity of the histogram distribution
Histogram Entropy	Measure of randomness or unpredictability of the histogram distribution
